# Does resistance training have an effect on levels of ferritin and atherogenic lipids in postmenopausal women? – *A pilot trial*

**DOI:** 10.1038/s41598-020-60759-z

**Published:** 2020-03-02

**Authors:** Liam J. Ward, Mats Hammar, Lotta Lindh-Åstrand, Emilia Berin, Hanna Lindblom, Marie Rubér, Anna-Clara Spetz Holm, Wei Li

**Affiliations:** 1 Department of Obstetrics and Gynaecology in Linköping, and Department of Biomedical and Clinical Sciences, Linköping University, Linköping, Sweden; 2 Occupational and Environmental Medicine Center in Linköping, and Department of Health, Medicine and Caring Sciences, Linköping University, Linköping, Sweden; 3 Department of Health, Medicine and Caring Sciences, Unit of Physiotherapy, Linköping University, Linköping, Sweden

**Keywords:** Randomized controlled trials, Cardiovascular diseases, Lifestyle modification

## Abstract

The objective of this study was to determine if 15 weeks of resistance training (RT) can alter the levels of blood lipids, body iron status, and oxidative stress in postmenopausal women with vasomotor symptoms. Postmenopausal women enrolled in a randomised controlled trial were allocated to either a sedentary control group (n = 29) or a RT group (n = 26). Blood samples were taken at week-0 and week-15 for all participants. Blood lipids and iron status were measured via routine clinical analyses. Immunoassays were used to measure oxidative stress markers. The RT group, with good compliance, was associated with significant reductions in ferritin, total cholesterol, low-density lipoprotein, and non-high-density lipoprotein cholesterol. Moreover, ferritin was positively correlated with atherogenic lipids while negatively correlated with high-density lipoprotein in RT women. This occurred without alterations in serum iron, transferrin, transferrin-saturation, C-reactive protein and oxidative stress markers. No differences were found in control women. This study suggests that RT in postmenopausal women both reduces levels of ferritin and counteracts atherogenic lipid profiles independent of an apparent oxidative mechanism. RT may be a beneficial intervention in postmenopausal women via an interaction between ferritin and lipids; however, further investigation in a larger cohort is essential.

## Introduction

The risk for cardiovascular disease (CVD) approximately doubles in women during the 10 years after menopause, and physical inactivity contributes to this risk^1,2^. The increase in CVD risk is partially attributed to the reduced levels of oestrogens (oestradiol and oestrone) that increase high-density lipoprotein (HDL) levels and decrease low-density lipoprotein (LDL)^3^. Oestrogens also increase production of the vasodilator nitric oxide by means of nitric oxide-synthase activation, and possess antioxidant properties reducing levels of reactive oxygen species^3^. Oxidative stress levels are a risk factor for CVD and known to be greater in postmenopausal women, compared to women of fertile age, with higher levels of 4-hydroxynonenal (HNE), malondialdehyde, and oxidised LDL^4^. In addition, postmenopausal women have an increased body iron burden, in comparison to premenopausal women, that can contribute to iron-driven oxidative stress^5^. Ferritin, an iron storage protein, is increased in postmenopausal women and associated with the metabolic syndrome, subclinical coronary atherosclerosis^6^, and carotid atherosclerosis in postmenopausal women^7^.

Physical inactivity increases the risk for the development of CVD and all-cause mortality in postmenopausal women^8^. A comparative study between sedentary postmenopausal women and postmenopausal women who perform regular aerobic exercise showed a significantly lower level of iron burden, including serum ferritin, iron and transferrin saturation, and oxidative stress levels in the active women^9^. Aerobic exercise has also shown beneficial effects on lipid profiles in postmenopausal women, with reduced levels of total cholesterol, LDL and triglyceride, and a simultaneous increase in HDL^10,11^. Resistance training (RT) is a form of anaerobic physical activity, whereby muscle actions are designed to improve muscular fitness. Moderate aerobic exercise and RT have both shown beneficial effect in lowering inflammatory activity^12^. However, it is unknown whether RT causes a correlative change in lipid profile, body iron status, and oxidative stress in postmenopausal women.

The aim of this study was to determine if a RT based 15 weeks regime of physical exercise can alter the blood lipid profile, body iron burden and oxidative stress in a cohort of postmenopausal women. The hypothesis for this study is that 15 weeks of RT in postmenopausal women will improve the blood lipid profile by reducing LDL and increasing HDL, and to reduce body iron burden and oxidative stress levels.

## Material and Methods

### Study design and population

This study was an analysis of secondary outcomes of a randomised controlled intervention trial (registered as ID: NCT01987778), with a published protocol^13^, conducted at Linköping University Hospital, Sweden. The research protocol was approved by the Regional Ethical Review Board in Linköping (2013/285-31 and 2013/338-32), and the study was performed in accordance to the Declaration of Helsinki and applicable sections of Good Clinical Practice. Written informed consent was obtained from all participants prior entry to the trial. The primary outcome of this trial was to assess the effect of RT on reducing hot flush frequency in postmenopausal women, and has recently been published including full details on the study recruitment, randomisation, sample size, and intervention^14^. In this sub-analysis, investigations into RT-associated effects on blood lipids, iron status, and oxidative stress were assessed.

**Figure 1:**
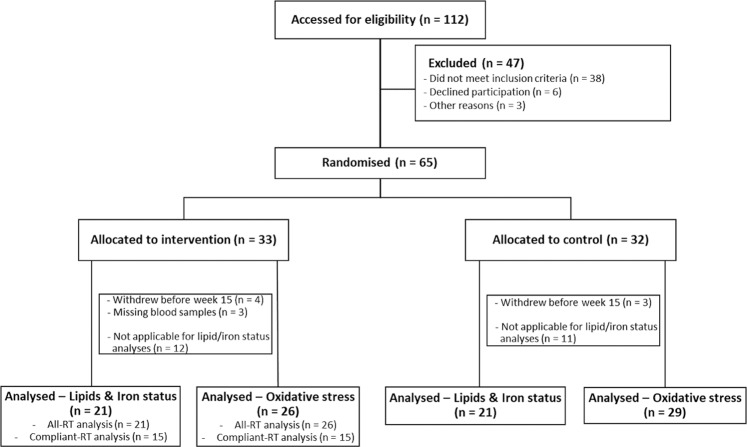
Study flow chart. Postmenopausal women were recruited and randomly allocated to the intervention (n = 33) or control group (n = 32). Blood lipid and iron status analyses were included into the trial after initiation, thus fewer samples were available for these analyses. Two series of analyses were perfomed in the resistance training (RT) intervention group, all-RT and compliant-RT, based on the participants good compliance to the resistance training regime.

Postmenopausal women (n = 65) with vasomotor symptoms, describing hot flashes/flushing and night sweats, were recruited and randomly assigned to either control (sedentary) or intervention (RT) groups for a study period of 15 weeks (Fig. 1). Block randomisation and allocation concealment was performed by an independent statistician, and allocations were blinded to researchers performing analyses until the conclusion of the study. Postmenopausal status was confirmed in all participants, and full inclusion/exclusion criteria have previously been published^13,14^, and described in the Supplementary Materials and Methods. Anthropometric parameters, age, body mass index (BMI), blood pressure, haemoglobin levels, were recorded at the start of the 15 weeks RT program for all participants and presented in Table 1. Relevant medication use was recorded (Table 1), the medication use represented stable usage that had been used by the women for at least three months prior entry to the trial (Table 1). Active smoking status was also recorded, with one smoking participant in each group (Table 1). No significant differences were recorded at baseline between the control and RT groups of postmenopausal women.

**Table 1:** Baseline anthropometric characteristics of study participants.

	Control (n = 29)	All-RT (n = 26)
Age, years (SD)	55.4 (5.0)	55.7 (5.1)
BMI, kg/m^2^ (SD)	26.7 (3.6)	28.1 (3.9)
Blood Pressure, mmHg (SD)		
Systolic	128.3 (16.6)	131.1 (14.0)
Diastolic	78.4 (10.1)	78.5 (7.1)
Haemoglobin, g/L (SD)	140.4 (7.8)	137.1 (9.9)
Medication used for, n (%)		
Hypertension	3 (10)	7 (27)
Rheumatoid arthritis	1 (3)	1 (4)
Hypothyroidism	2 (7)	3 (12)
Crohn’s disease	1 (3)	1 (4)
Smoking, n (%)	1 (3)	1 (4)

There were no significant differences between groups in any of the variables.

RT – resistance training; SD – standard deviation.

### Intervention

The RT regime was comprised of 15 weeks structured RT, targeting all major muscles groups, performed three times per week. The regime consisted of six exercises in seated resistance machines and two body-weight exercises in the following order; chest press, leg press, seated row, leg curl, latissimus dorsi pull-down, leg extension, crunches and back raises. The seated exercises were performed in 15–20 repetitions in two sets week 1–3, and 8–12 repetitions in two sets week 4–15. Body-weight exercises were performed until exhaustion in two sets^13,14^. A physiotherapist instructed every woman at baseline and supervised one session per week to assess fidelity to the training schedule, which was adapted to each individual with gradually increased loads during the trial.

Personal physical activity logs, together with logs from the electronic card system at the designated gym, were recorded to track compliance. Established study criteria stipulated good compliance as an average of two or more RT sessions per week, excluding time-off due to illness^13^. Participants in the control group were instructed to remain sedentary, and not change their level of physical activity for the study period.

A total of 58 women completed the study, 29 controls and 29 in the RT group, with seven women being excluded due to withdrawal criteria or lack of compliance. In addition, plasma samples were missing for another three women in the RT group, giving a total of 26 in RT group. Within the RT group, the number of participants with good compliance to the RT protocol was 15 out of 26 women (58%), whereas 11 women had an average of below two RT sessions per week. The average number of RT sessions for all-RT, compliant-RT and non-compliant-RT women were 2.2, 2.6 and 1.7 sessions per week, respectively.

Fasting blood samples were taken at week-0 and again at week-15 of the study period for each participant. Within 1 hour of collection blood samples were centrifuged, 10 minutes at 1500 G, and plasma (EDTA) was immediately frozen and stored at −70 °C for later analysis of oxidative stress markers. Fasting blood samples, for a subset of participants, were also taken for direct analysis of blood lipids and iron status.

### Blood lipids and iron status

A subset of participants (n = 21 for both control and RT groups) were available for analysis of blood lipids and iron status. Plasma was used for the analysis of blood lipids and iron status, including; cholesterol, LDL, HDL, triglycerides, apolipoprotein A1, apolipoprotein B, ferritin, iron, transferrin, and transferrin saturation. All analyses were performed via routine clinical analysis in an accredited laboratory (ISO/IEC 17025; Diagnostikcentrum i Östergötland, Linköping, Sweden). Methodological principles are described in the Supplementary Materials and Methods.

### Oxidative stress markers

Total antioxidant capacity (TAC) was measured using the OxiSelect TAC Assay Kit (Cell Biolabs, San Diego, CA, USA). This assay measures the capacity of biomolecules within a sample to neutralise radicals, via a single electron transfer mechanism, based on the reduction of copper (II) to copper (I). The assay was performed according to manufacturer guidelines. Briefly, 20 µL of plasma samples were added to a 96-well plate. Initial absorbance was then measured at 490 nm. Copper (II) ions were then added to initiate the reaction, and after 2 minutes the absorbance was measured again at 490 nm. TAC was then determined by comparison with a set of uric acid standards.

HNE, a natural by-product of lipid peroxidation, was assayed using the OxiSelect HNE Adduct Competitive ELISA Kit (Cell Biolabs) according to manufacturer guidelines. Briefly, 50 µL of plasma samples were added to HNE conjugate coated 96-well plates and incubated at room temperature for 10 minutes. Antibody probing was performed using primary anti-HNE antibody, followed by secondary HRP-conjugated antibody, both of which were incubated sequentially at room temperature for one hour. Absorbance was measured spectrophotometrically at a wavelength of 450 nm. HNE concentration was determined using a standard curve of predetermined HNE-bovine serum albumin samples.

Protein carbonyl derivatives, common products of protein oxidation, were assayed using the OxiSelect Protein Carbonyl ELISA Kit (Cell Biolabs) according to manufacturer guidelines. Briefly, plasma samples containing 10 µg/mL of protein were absorbed onto a 96-well plate over two hours at 37 °C. Protein carbonyls in the samples were then derivatised to 2,4-dinitrophenylhydrazine (DNPH) via incubation at room temperature in the dark for 45 minutes. Antibody probing was performed using primary anti-DNP antibody, followed by secondary HRP-conjugated antibody, both of which were incubated sequentially at room temperature for 1 hour. Absorbance was measured spectrophotometrically at a wavelength of 450 nm. Protein carbonyl concentration was determined using a standard curve of predetermined reduced and oxidised bovine serum albumin samples.

### Statistics

Data are expressed as either mean ± standard deviation (SD) or median ± quartile range, dependent on data distribution, unless otherwise stated. All statistical analyses were performed in SPSS v.24.0 (IBM, Portsmouth, UK). Two series of statistical analyses were performed, based on: (1) results from all RT participants who had measurements fom the baseline to 15 weeks regardless of the level of compliance (herein termed “*all-RT* ”), and (2) results from only RT participants with good compliance (herein termed “*compliant-RT* ”). Data comparisons between week-0 and week-15 in either control or RT groups were performed using non-parametric Wilcoxon-signed rank statistics. Comparisons between control and RT groups after 15 weeks were performed using non-parametric Mann-Whitney *U* test, by using a percentage of the week-15 values to the corresponding week-0 values (percentage change from baseline). Correlations between ferritin and blood lipids were calculated using Spearman’s correlation test. Correlations were run for both time-points individually, either week-0 or week-15, and also for values representing the change across the study period (week-15 minus week-0). A *p*-value < 0.05 was considered significant.

## Results

### RT resulted in significantly reduced levels of atherogenic lipids

A subset of participants was available for blood lipids and iron status analysis (n = 21 for both control and RT groups). No significant changes were observed from week-0 to week-15 regarding any of the lipid parameters in either control or in all-RT group (Table 2). However, significant decreases were observed in the compliant-RT group in total cholesterol, LDL, and non-HDL cholesterol (Table 2). At week-0, no statistical differences were seen between the groups (Supplementary Table S1). Among the non-complaint-RT women, there was no significant differences in lipid profiles (Supplementary Table S2).

**Table 2:** Blood lipids and body iron parameters measured at 0-weeks and at 15-weeks resistance training (RT).

	Control (n = 21)		P-value	All-RT (n = 21)		P-value	Compliant-RT (n = 15)		P-value
	0 weeks	15 weeks		0 weeks	15 weeks		0 weeks	15 weeks	
TC (mmol/L)	6.2 (5.7–6.8)	6.0 (5.5–7.0)	0.66	6.0 (5.1–6.7)	5.8 (4.6–6.8)	0.13	5.6 (4.8–6.6)	5.3 (4.4–6.5)	0.03*
LDL (mmol/L)	3.6 (3.2–4.2)	3.8 (3.0–4.3)	0.93	3.4 (2.7–4.1)	3.1 (2.4–4.3)	0.24	2.9 (2.6–4.1)	2.9 (2.4–4.1)	0.04*
HDL (mmol/L)	1.8 (1.6–2.5)	2.2 (1.5–2.6)	0.98	1.9 (1.6–2.2)	2.0 (1.6–2.4)	0.86	1.8 (1.5–2.2)	1.8 (1.6–2.4)	0.97
TG (mmol/L)	0.9 (0.7–1.4)	0.9 (0.8–1.6)	0.47	1.0 (0.7–1.6)	1.2 (0.8–1.4)	0.42	1.0 (0.7–1.7)	1.2 (0.8–1.4)	0.18
Non-HDL (mmol/L)	4.1 (3.6–4.8)	4.3 (3.4–4.9)	0.84	3.8 (3.1–4.8)	3.5 (2.9–4.9)	0.10	3.3 (3.0–5.1)	3.2 (2.8–4.8)	0.02*
Apo-A1 (g/L)	1.8 (1.5–2.0)	1.7 (1.6–2.0)	0.85	1.8 (1.6–2.0)	1.7 (1.6–2.0)	0.87	1.8 (1.6–2.0)	1.7 (1.5–1.9)	0.21
Apo- B (g/L)	1.2 (1.0–1.3)	1.1 (0.9–1.2)	0.72	1.1 (0.9–1.3)	1.1 (0.9–1.3)	0.13	1.0 (0.8–1.3)	1.0 (0.8–1.3)	0.07
Ferritin (µg/L)	120 (61–163)	96 (68–157)	0.55	100 (51–147)	86 (44–139)	0.01*	70 (42–141)	65 (18–118)	0.02*
Iron	17 (14–19)	16 (14–20)	0.72	17 (13–21)	17 (14–21)	0.71	18 (12–24)	17 (15–22)	0.82
TF	2.5 (2.3–2.75)	2.6 (2.3–2.8)	0.95	2.5 (2.3–2.7)	2.6 (2.3–2.8)	0.84	2.6 (2.3–2.7)	2.7 (2.2–2.9)	0.93
TF-saturation (%)	27 (20–31)	26 (21–33)	0.98	29 (23–34)	26 (21–33)	0.17	31 (23–36)	26 (22–36)	0.23

Wilcoxon-signed rank tests was used to compare measured parameters across the 15-week study period.

Values are median (quartile range: Q1–Q3); values to be considered significant are displayed in bold text, with *p < 0.05.

Apo – apolipoprotein; HDL – high-density lipoprotein; LDL – low-density lipoprotein; RT – resistance training; TC – total cholesterol; TF – transferrin; TG – triglycerides.

### RT significantly reduced the levels of plasma ferritin

**Figure 2:**
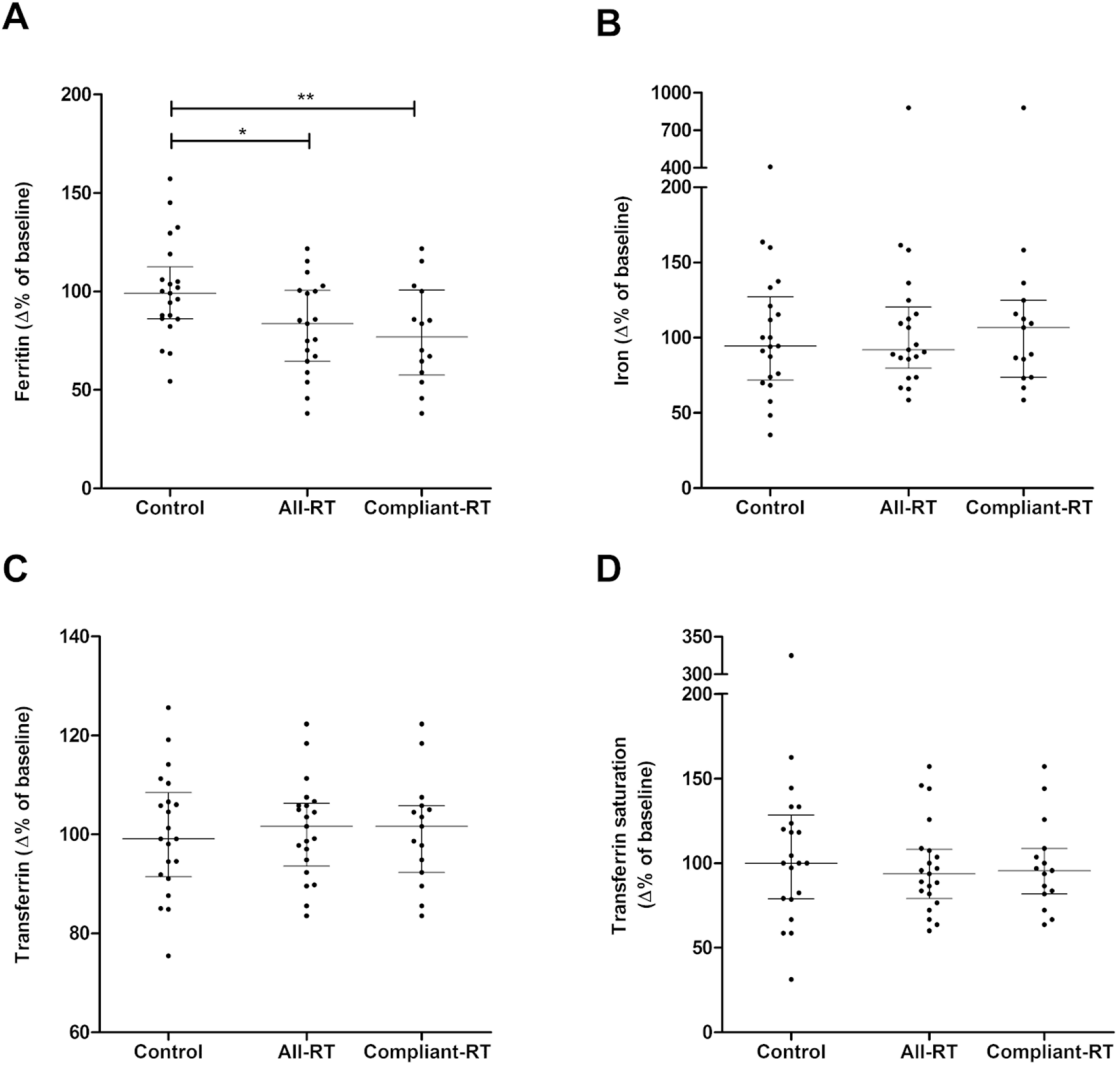
15-weeks resistance training (RT) was associated with decreases in plasma ferritin levels but not the other iron status parameters in postmenopausal women. Postmenopausal women were randomised into either control (n = 21) or RT groups, presented as both all-RT (n = 21) and compliant-RT (n = 15). Body iron burden parameters were measured at week-0 and week-15 of the study period. Values were presented as percentage of corresponding week-0 values, median ± IQR. (**A**) ferritin. (**B**) iron, (**C**) transferrin and (**D**) transferrin saturation.

Ferritin decreased significantly between week-0 and week-15 in the all-RT group (p = 0.01) and compliant-RT groups (p = 0.02, Table 2). Additionally, ferritin levels decreased significantly more in the all-RT and compliant-RT groups when compared to the control group (Fig. 2A) using values normalised to baseline (% of corresponding week-0 values). No significant differences were present for iron, transferrin or transferrin saturation between controls and RT groups at 0-weeks or 15-weeks or regarding change over the 15 weeks (Table 2 and Fig. 2B–D). There was no statistical difference in ferritin levels between control and RT groups at week-0 (Supplementary Table S1). Among the non-compliant-RT women, no significant differences were observed (Supplementary Table S2). Additionally, CRP levels were not found to differ significantly in all groups after 15 weeks (8.7 µg/mL for control, 8.6 µg/mL for all-RT, and 9.3 µg/mL for compliant-RT group).

### RT did not affect oxidative stress markers

The complete study cohort was available for oxidative stress analysis (n = 29 for control and n = 26 for RT). To compare controls with RT group after 15 weeks, the week-15 values were presented as a percentage of the corresponding 0-weeks values. We found no statistical difference between study groups in TAC, HNE or protein carbonyl (Supplementary Fig. S1).

### Ferritin positively correlated with atherogenic lipid profiles and negatively correlated with HDL in RT women

Correlation analyses between ferritin and blood lipids were performed for control and both RT groups. Significant negative correlations were found between ferritin and HDL, in the all-RT group at week-0, and in all participant groups when analysing the change between week-0 and week-15 (Table 3). In the all-RT group, there were significant positive correlations between ferritin and triglycerides, non-HDL, and apolipoprotein-B when analysing the change between week-0 and week-15 (Table 3).

**Table 3:** Correlation analyses of ferritin and lipid parameters measured in control and resistance training (RT) groups.

Parameters	Week-0			Week-15			Change (week-15–week-0)		
	Control (n = 21)	All-RT (n = 21)	Compliant-RT (n = 15)	Control (n = 21)	All-RT (n = 21)	Compliant-RT (n = 15)	Control (n = 21)	All-RT (n = 21)	Compliant-RT (n = 15)
Ferritin & TC	−0.25	0.20	0.06	−0.16	0.22	−0.13	−0.04	0.11	−0.15
Ferritin & LDL	−0.17	0.19	−0.08	−0.30	0.20	−0.04	0.08	0.36	0.17
Ferritin & HDL	0.12	−0.47*	−0.45	0.17	−0.34	−0.35	−0.63**	−0.49*	−0.56*
Ferritin & TG	−0.31	0.52*	0.56*	−0.09	0.42	0.24	−0.13	0.52*	0.48
Ferritin & non-HDL	−0.23	0.24	0.01	−0.29	0.24	0.01	0.07	0.48*	0.32
Ferritin & Apo-A1	0.10	−0.31	−0.22	0.17	−0.23	−0.41	−0.31	−0.15	−0.38
Ferritin & Apo-B	−0.36	0.18	−0.07	−0.21	0.22	−0.08	−0.21	0.52*	0.27

Values are Spearman correlation coefficients (ρ).

Significant correlations are displayed in bold text, with *p < 0.05 and **p < 0.01.

Apo – apolipoprotein; HDL – high-density lipoprotein; LDL – low-density lipoprotein; RT – resistance training; TC – total cholesterol; TG – triglycerides.

## Discussion

Physical activity interventions are known to reduce the risk of developing CVD in postmenopausal women, likely by altering lipid profiles or improving antioxidant capacity and decreasing body iron burden^9–11^. However, it is unclear whether RT in postmenopausal women can lead to correlative changes in iron metabolism, lipid profile, and oxidative stress, which have been separately linked to the ferritin related cardiometabolic conditions. Our study shows that 15 weeks of RT not only significantly reduced the levels of ferritin, but also levels of total cholesterol, LDL cholesterol, and non-HDL cholesterol. Moreover, among RT women correlative changes were found between ferritin levels and atherogenic lipid profiles.

Physical inactivity and onset of menopause are known risk factors for CVD in women^15,16^. It has previously been shown that exercise with 10 weeks Nordic walking or a 3-month high-intensity aerobic training have beneficial effects on lipid profiles amongst postmenopausal women^10,11^. The majority of RT trials in postmenopausal women have primarily investigated overweight/obese postmenopausal women, and results regarding lipid profiles are contradictory with some trials showing the benefits of RT^17^, whilst others show no benefit^18^ or no additional benefit in combined aerobic and RT trials^19^. The present study demonstrated that 15 weeks of RT with good compliance had beneficial effects on lipid profiles of total cholesterol, LDL cholesterol, and non-HDL cholesterol in the overall population.

Ferritin has been associated with several cardiometabolic conditions including dyslipidaemia, hypertension, obesity, and insulin resistance, although the mechanisms by which they are associated are still not fully understood. Ferritin is an iron storage protein and increased serum ferritin levels have been associated with onset of coronary heart disease^20^, myocardial infarction, and coronary artery disease related mortality^21^. High levels of serum ferritin was found to be associated with increased risk of stroke in postmenopausal women^22^, as well as with metabolic syndrome^23^. In addition, ferritin is also recognised as an acute phase marker protein and is potentially higher during infection or inflammation. Ferritin was found to be positively associated with CRP in patients with type 2 diabetes^24^. In the present study, we found that RT trained women had reduced levels of ferritin, but no change in other parameters associated with iron metabolism (iron, transferrin and transferrin saturation), nor in the levels of CRP and oxidative stress markers. This may suggest that the RT-associated reduction in ferritin levels are unrelated to its function in iron metabolism or oxidative stress mechanisms. However, RT-associated effects on low grade inflammation via lowering ferritin cannot be excluded and warrant further study.

Ferritin levels have been associated with atherogenic lipid profiles in previous studies. It has been reported a negative correlation between levels of serum ferritin and HDL-cholesterol, while positive correlations between levels of serum ferritin and total cholesterol, triglyceride, and LDL-cholesterol^25^. In the present study, for the first time, we demonstrate that ferritin in postmenopausal women participating in RT over 15 weeks was positively correlated with atherogenic lipids including triglycerides, non-HDL and apolipoprotein-B, while negatively correlated with HDL. These results may indicate a mechanistic link between RT mediated changes of iron and lipid metabolisms among postmenopausal women.

The molecular mechanism behind the association between increased ferritin and metabolic syndrome and CVD is not yet fully understood. A possible mechanism is that iron serves as a catalyst for free radical reactions in oxidative stress in atherogenesis and metabolic syndrome^26^. Moderate exercise and an active lifestyle have been demonstrated to be beneficial for not only prevention of oxidative stress but also protection of cardiovascular related morbidity^27^, type II diabetes, metabolic syndrome^28^, and Alzheimer’s disease^29^. However, inconsistent findings have been reported frequently in the area. A recent study showed that both aerobic exercise and RT can increase lipid peroxidation^30^. In contrast, another study on aerobic exercise documented a decrease in lipid peroxidation^31^. In the present study, we found no significant difference in levels of studied markers of oxidative stress after 15 weeks RT exercise. This suggests that effect of RT seen in the present study may depend on other functions of ferritin in the development of cardiometabolic disorders, possibly energy metabolism^7^. However, the muscle specific response could be different. It is known that skeletal muscle fibres produce free-radicals during exercise^32^. Previous correlations between malondialdehyde, a pro-oxidative stress marker, and RT-induced changes in skeletal muscle have been seen in healthy older women^33^. Thus, the oxidative stress mechanisms in the local muscle environment should not be ruled out, and be considered for future studies.

There are limitations in the current study that need to be considered. The primary outcome for this trial was to investigate the effects of RT on vasomotor symptoms in postmenopausal women, and as such power calculations were based on this primary outcome. Thus, the investigations into RT-associated effects on iron metabolism, oxidative stress, and lipid profiles were not the primary outcome in this clinical cohort, rather a pilot clinical trial. Therefore, the participant number included is relatively low. Participants’ dietary intake were not recorded that may affect our results, particularly on the oxidative stress markers with respect to levels of iron and antioxidants consumed. Thus, additional studies with a larger cohort including recording of dietary intake of iron and antioxidants are warranted.

## Conclusions

The present study shows that postmenopausal women who engaged in supervised RT over a 15-week period had significantly lowered levels of ferritin and, with good compliance, atherogenic lipids. The correlations of ferritin with atherogenic lipids or HDL may suggest a mechanistic link between lipid and ferritin function mediated by RT in postmenopausal women. The RT-associated changes in ferritin levels were not associated with changes in iron, CRP and oxidative stress levels. The results indicate that RT in postmenopausal women may have beneficial outcomes in preventing atherosclerosis and reducing CVD risk. However, these findings need to be confirmed in a large-scale cohort.

## Supplementary information

Supplementary information.

## Data Availability

All data are summarised in this published article, and the raw datasets are available from the corresponding authors or reasonable request.

## References

[1] Lisabeth L, Bushnell C (2012). Menopause and Stroke: An Epidemiologic Review. Lancet neurol..

[2] Mosca L (2011). Effectiveness-Based Guidelines for the Prevention of Cardiovascular Disease in Women—2011 Update. Circulation.

[3] Stice JP, Lee JS, Pechenino AS, Knowlton AA (2009). Estrogen, aging and the cardiovascular system. Future cardiol..

[4] Signorelli SS (2006). Behaviour of some indicators of oxidative stress in postmenopausal and fertile women. Maturitas.

[5] Yuan XM, Li W (2003). The iron hypothesis of atherosclerosis and its clinical impact. Ann. Med..

[6] Seo SK (2015). Association of serum ferritin levels with metabolic syndrome and subclinical coronary atherosclerosis in postmenopausal Korean women. Clin. Chim Acta.

[7] Ma H (2015). Serum ferritin levels are associated with carotid atherosclerosis in Chinese postmenopausal women: the Shanghai Changfeng Study. Br. J. Nutr..

[8] Oguma Y, Sesso H, Paffenbarger R, Lee I (2002). Physical activity and all cause mortality in women: a review of the evidence. Br. J. Sports Med..

[9] Bartfay W, Bartfay E (2013). A Case–Control Study Examining the Effects of Active Versus Sedentary Lifestyles on Measures of Body Iron Burden and Oxidative Stress in Postmenopausal Women. Biol. Res. Nurs..

[10] Hagner-Derengowska M (2015). Effects of Nordic Walking and Pilates exercise programs on blood glucose and lipid profile in overweight and obese postmenopausal women in an experimental, nonrandomized, open-label, prospective controlled trial. Menopause.

[11] Mandrup CM (2017). Effects of high-intensity training on cardiovascular risk factors in premenopausal and postmenopausal women. Am. J. Obstet. Gynecol..

[12] Soares FHR, de Sousa MBC (2013). Different Types of Physical Activity on Inflammatory Biomarkers in Women With or Without Metabolic Disorders: A Systematic Review. Women & Health.

[13] Berin E, Hammar ML, Lindblom H, Lindh-Åstrand L, Spetz Holm A-CE (2016). Resistance training for hot flushes in postmenopausal women: Randomized controlled trial protocol. Maturitas.

[14] Berin E (2019). Resistance training for hot flushes in postmenopausal women: A randomised controlled trial. Maturitas.

[15] Humphries KH (2017). Sex differences in cardiovascular disease – Impact on care and outcomes. Front. Neuroendocrinol..

[16] Elhakeem A (2018). Physical Activity, Sedentary Time, and Cardiovascular Disease Biomarkers at Age 60 to 64 Years. J. Am. Heart Assoc..

[17] Wooten JS (2011). Resistance Exercise and Lipoproteins in Postmenopausal Women. Int. J. Sports Med..

[18] Mohanka M (2006). Serum Lipoproteins in Overweight/Obese Postmenopausal Women: A One-Year Exercise Trial. Med. Sci. Sports Exerc..

[19] Rossi FE (2016). Combined Training (Aerobic Plus Strength) Potentiates a Reduction in Body Fat but Demonstrates No Difference on the Lipid Profile in Postmenopausal Women When Compared With Aerobic Training With a Similar Training Load. J. Strength Cond. Res..

[20] Ahluwalia N (2010). Iron Status Is Associated with Carotid Atherosclerotic Plaques in Middle-Aged Adults. J. Nutr..

[21] Salonen JT (1992). High stored iron levels are associated with excess risk of myocardial infarction in eastern Finnish men. Circulation.

[22] van der DL (2005). Serum Ferritin Is a Risk Factor for Stroke in Postmenopausal Women. Stroke.

[23] Jehn M, Clark JM, Guallar E (2004). Serum Ferritin and Risk of the Metabolic Syndrome in U.S. Adults. Diabetes Care.

[24] Alam F, Fatima F, Orakzai S, Iqbal N, Fatima SS (2014). Elevated levels of ferritin and hs-CRP in type 2 diabetes. J. Pak. Med. Assoc..

[25] Ellidag, H. Y. *et al*. The relationship between serum ferritin levels and serum lipids and HDL function with respect to age and gender. *Ukr. Biochem. J*. **88**, 76–85 (2016). 10.15407/ubj88.06.076 29236376

[26] Yuan X-M, Li W (2008). Iron Involvement in Multiple Signaling Pathways of Atherosclerosis: A Revisited Hypothesis. Curr. Med. Chem..

[27] Orkaby AR, Forman DE (2018). Physical activity and CVD in older adults: an expert’s perspective. Expert Rev. Cardiovasc. Ther..

[28] Church T (2011). Exercise in Obesity, Metabolic Syndrome, and Diabetes. Progress in Cardiovasc. Dis..

[29] DeWeerdt S (2011). Prevention: Activity is the best medicine. Nature.

[30] Carteri RB (2016). Acylated Ghrelin and Circulatory Oxidative Stress Markers Responses to Acute Resistance and Aerobic Exercise in Postmenopausal Women. J. Phys. Act. Health.

[31] Karolkiewicz J (2009). Response of oxidative stress markers and antioxidant parameters to an 8-week aerobic physical activity program in healthy, postmenopausal women. Arch. Gerontol. Geriatr..

[32] Bailey DM (2007). Electron paramagnetic spectroscopic evidence of exercise-induced free radical accumulation in human skeletal muscle. Free Radic. Res..

[33] Carru C (2018). Markers of oxidative stress, skeletal muscle mass and function, and their responses to resistance exercise training in older adults. Exp. Gerontol..

