# Supplementary material for: Does resistance training have an effect on levels of ferritin and atherogenic lipids in postmenopausal women? – A pilot trial
Source: Sci Rep. 2020 Mar 2;10:3838. doi: 10.1038/s41598-020-60759-z (PMC7052219; doi:10.1038/s41598-020-60759-z)
Supplement: Supplementary file 1 — Supplementary information. [file 41598_2020_60759_MOESM1_ESM.pdf]

Does resistance training have an effect on levels of ferritin and atherogenic lipids in postmenopausal women? – *A pilot trial*

Liam J. Ward[a,b]*, Mats Hammar[a], Lotta Lindh-Åstrand[a], Emilia Berin[a], Hanna Lindblom[c], Marie Rubér[a], Anna-Clara Spetz Holm[a], Wei Li[a]*

List of supplementary material

Supplementary Material and Methods.

Supplementary Table S1. Baseline (week-0) comparisons for blood lipids, iron status, and oxidative stress parameters measured in postmenopausal women.

Supplementary Table S2. Blood lipids, iron status, and oxidative stress parameters measured at 0-weeks and at 15-weeks for women deemed non-compliant to the RT training regime.

Supplementary Figure S1. The effect of 15-weeks resistance training (RT) on oxidative stress in postmenopausal women.

1. Supplementary materials and methods

1.1. Inclusion and exclusion criteria for study participants

Postmenopausal women, with status confirmed as; women ≥ 45 years old with ≥ 12 months amenorrhea since last natural menstrual period, or women with amenorrhea due to hysterectomy or an intrauterine device if follicle-stimulating hormone (FSH) levels were > 20 mIU/mL. In addition, women must have been in good general health with physical ability to participate in RT regime, and given signed informed consent. Exclusion criteria included; > 75 minutes per week of moderate- to vigorous-intensity physical activity, > 225 minutes per week of any physical activity, capillary haemoglobin < 110 g/L, blood pressure systolic > 160 mmHg or diastolic > 100 mmHg, systemic hormone therapy, antidepressants were allowed if the dose was stable and treatment unrelated to vasomotor symptoms, and medical conditions limiting full participation in RT.

1.2. Clinical laboratory analysis of blood lipids and iron status

The methodological principle used for the analyses of cholesterol, HDL, LDL, non-HDL and triglycerides was enzymatic colourimetric analysis. Photometric measurements were taken at 700/505 nm for cholesterol, LDL, non-HDL and triglycerides, and at 700/600 nm for HDL, using the Cobas c501/701 post-analytical system (Roche Diagnostics Scandinavia AB, Solna, Sweden).

The methodological principle for the analyses of apolipoprotein A1, apolipoprotein B, transferrin and transferrin saturation was immunoturbimetric analysis. Absorbance measurements were taken at 700/340 nm for apolipoprotein A1 and apolipoprotein B, and at 700/505 nm for transferrin and transferrin saturation, using the Cobas c501/701 post-analytical system.

Iron analysis was based on the FerroZine method without deproteinisation, and colourimetric measurements taken at 700/600 nm using the Cobas c501/701 post-analytical system.

Ferritin analysis was performed via sandwich immunoassay with ruthenium labelling, chemiluminescent emission was measured at 620 nm using the Cobas e602 system (Roche Diagnostics Scandinavia AB).

All methods performed in this laboratory are routinely subjected to both internal and external quality control.

Supplementary Table S1. Baseline (week-0) comparisons for blood lipids, iron status, and oxidative stress parameters measured in postmenopausal women. Mann-Whitney *U* tests were used to compare control and all-RT values, and control and compliant-RT values.

	**Control (n = 21)**	**All-RT (n = 21)**	**P-value**	**Compliant-RT (n = 15)**	**P-value**
TC (mmol/L)	6.2 (5.7-6.8)	6.0 (5.1-6.7)	0.37	5.6 (4.8-6.6)	0.23
LDL (mmol/L)	3.6 (3.2-4.2)	3.4 (2.7-4.1)	0.25	2.9 (2.6-4.1)	0.11
HDL (mmol/L)	1.8 (1.6-2.5)	1.9 (1.6-2.2)	0.78	1.8 (1.5-2.2)	0.63
TG (mmol/L)	0.9 (0.7-1.4)	1.0 (0.7-1.6)	0.69	1.0 (0.7-1.7)	0.48
Non-HDL (mmol/L)	4.1 (3.6-4.8)	3.8 (3.1-4.8)	0.30	3.3 (3.0-5.1)	0.19
Apo-A1 (g/L)	1.8 (1.5-2.0)	1.8 (1.6-2.0)	0.87	1.8 (1.6-2.0)	0.96
Apo- B (g/L)	1.2 (1.0-1.3)	1.1 (0.9-1.3)	0.39	1.0 (0.8-1.3)	0.28
Ferritin (µg/L)	120 (61-163)	100 (51-147)	0.53	70 (42-141)	0.17
Iron	17 (14-19)	17 (13-21)	0.83	18 (12-24)	0.61
TF	2.5 (2.3-2.75)	2.5 (2.3-2.7)	0.91	2.6 (2.3-2.7)	0.66
TF-saturation (%)	27 (20-31)	29 (23-34)	0.24	31 (23-36)	0.21
	**Control (n = 29)**	**All-RT (n = 26)**		**Compliant-RT (n = 15)**	
P. Carbonyl (nmol/g)	499.7 (403.9-708.4)	394.5 (46.9-635.0)	0.16	441.4 (46.9-735.2)	0.46
4-HNE (µg/mL)	7.9 (0.8-18.3)	10.0 (0.8-20.7)	0.76	16.8 (3.7-20.9)	0.19
TAC (CRE)	299.0 (238.7-358.2)	336.1 (258.8-382.5)	0.34	336.1 (280.4-385.7)	0.30

Values are median (quartile range: Q1-Q3).

Apo – apolipoprotein; HDL – high-density lipoprotein; LDL – low-density lipoprotein; P. – protein; RT – resistance training; TAC – total antioxidant capacity; TC – total cholesterol; TF – transferrin; TG – triglycerides; 4-HNE – 4-hydroxynonenal.

Supplementary Table S2. Blood lipids, iron status, and oxidative stress parameters measured at 0-weeks and at 15-weeks for women deemed non-compliant to the RT training regime. Wilcoxon-signed rank tests was used to compare measured parameters across the 15-week study period.

	**Non-compliant-RT (n = 6)**		**P-value**
	**0 weeks**	**15 weeks**	
TC (mmol/L)	6.3 (5.1-7.4)	7.0 (5.4-7.6)	0.83
LDL (mmol/L)	3.6 (3.1-4.6)	4.3 (2.9-4.7)	0.56
HDL (mmol/L)	2.1 (1.5-2.9)	2.2 (1.8-2.4)	0.83
TG (mmol/L)	0.8 (0.6-1.7)	1.1 (0.9-1.5)	0.68
Non-HDL (mmol/L)	4.1 (3.4-5.3)	4.8 (3.3-5.3)	0.68
Apo-A1 (g/L)	1.9 (1.5-2.0)	2.0 (1.7-2.1)	0.19
Apo- B (g/L)	1.1 (1.0-1.5)	1.2 (0.9-1.4)	0.83
Ferritin (µg/L)	119 (93-263)	102 (86-274)	0.50
Iron	16 (12-21)	17 (10-21)	0.40
TF	2.3 (2.2-2.6)	2.4 (2.3-2.6)	0.84
TF-saturation (%)	29 (23-31)	26 (18-32)	0.44
	**Non-compliant-RT (n = 11)**		
P. Carbonyl (nmol/g)	355.1 (125.0-618.6)	532.3 (278.1-660.6)	0.06
4-HNE (µg/mL)	1.7 (0.8-10.5)	1.7 (0.8-8.3)	0.16
TAC (CRE)	336.1 (237.1-359.2)	283.2 (221.7-319.5)	0.28

Values are median (quartile range: Q1-Q3).

Apo – apolipoprotein; HDL – high-density lipoprotein; LDL – low-density lipoprotein; P. – protein; RT – resistance training; TAC – total antioxidant capacity; TC – total cholesterol; TF – transferrin; TG – triglycerides; 4-HNE – 4-hydroxynonenal.

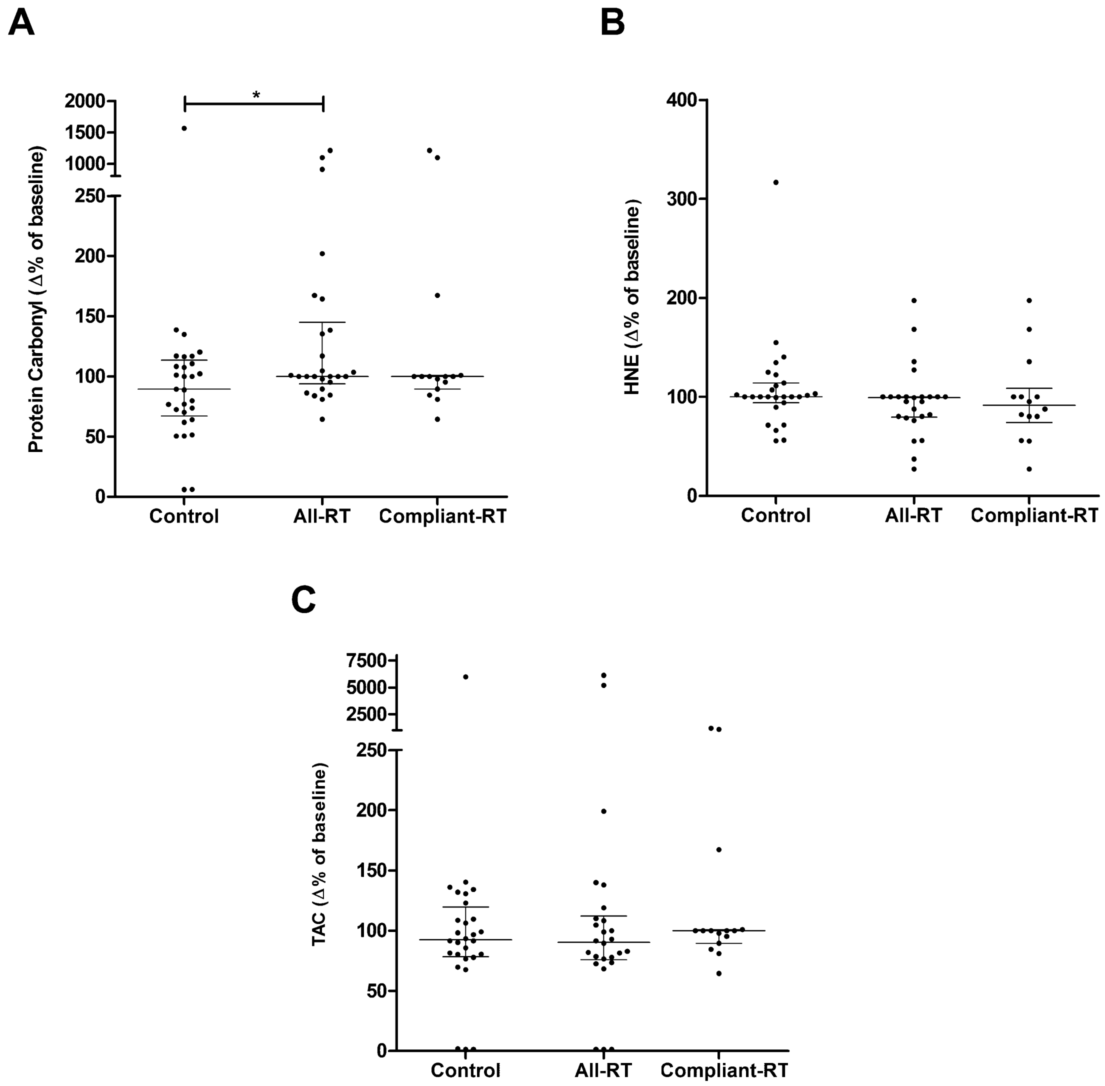

Supplementary Figure 1. The effect of 15-weeks resistance training (RT) on oxidative stress in postmenopausal women. Postmenopausal women were randomised into either control (n = 29) or RT groups, presented as both all-RT (n = 26) and compliant-RT (n = 15). Oxidative stress markers were measured at week-0 and week-15 of the study period. Values were normalised to baselines values (% of corresponding week-0 values). (A) Protein carbonyl, (B) 4-hydroxynonenal (HNE), and (C) total antioxidant capacity (TAC).
